# Reconciling Egg- and Antigen-Based Estimates of *Schistosoma mansoni* Clearance and Reinfection: A Modeling Study

**DOI:** 10.1093/cid/ciab679

**Published:** 2021-08-06

**Authors:** Jessica Clark, Arinaitwe Moses, Andrina Nankasi, Christina L Faust, Adriko Moses, Diana Ajambo, Fred Besigye, Aaron Atuhaire, Aidah Wamboko, Lauren V Carruthers, Rachel Francoeur, Edridah M Tukahebwa, Joaquin M Prada, Poppy H L Lamberton

**Affiliations:** 1 Wellcome Centre for Integrative Parasitology, Institute of Biodiversity, Animal Health & Comparative Medicine, University of Glasgow, Glasgow, United Kingdom; 2 Vector Control Division, Ministry of Health, Uganda; 3 Faculty of Science & Engineering, University of Chester, Chester, United Kingdom; 4 Faculty of Health & Medical Sciences, University of Surrey, Guildford, United Kingdom

**Keywords:** Diagnostics, temporal dynamics, POC-CCA, G-Score, Kato-Katz

## Abstract

**Background:**

Despite decades of interventions, 240 million people have schistosomiasis. Infections cannot be directly observed, and egg-based Kato-Katz thick smears lack sensitivity, affected treatment efficacy and reinfection rate estimates. The point-of-care circulating cathodic antigen (referred to from here as POC-CCA+) test is advocated as an improvement on the Kato-Katz method, but improved estimates are limited by ambiguities in the interpretation of trace results.

**Method:**

We collected repeated Kato-Katz egg counts from 210 school-aged children and scored POC-CCA tests according to the manufacturer’s guidelines (referred to from here as POC-CCA+) and the externally developed G score. We used hidden Markov models parameterized with Kato-Katz; Kato-Katz and POC-CCA+; and Kato-Katz and G-Scores, inferring latent clearance and reinfection probabilities at four timepoints over six-months through a more formal statistical reconciliation of these diagnostics than previously conducted. Our approach required minimal but robust assumptions regarding trace interpretations.

**Results:**

Antigen-based models estimated higher infection prevalence across all timepoints compared with the Kato-Katz model, corresponding to lower clearance and higher reinfection estimates. Specifically, pre-treatment prevalence estimates were 85% (Kato-Katz; 95% CI: 79%–92%), 99% (POC-CCA+; 97%–100%) and 98% (G-Score; 95%–100%). Post-treatment, 93% (Kato-Katz; 88%–96%), 72% (POC-CCA+; 64%–79%) and 65% (G-Score; 57%–73%) of those infected were estimated to clear infection. Of those who cleared infection, 35% (Kato-Katz; 27%–42%), 51% (POC-CCA+; 41%–62%) and 44% (G-Score; 33%–55%) were estimated to have been reinfected by 9-weeks.

**Conclusions:**

Treatment impact was shorter-lived than Kato-Katz–based estimates alone suggested, with lower clearance and rapid reinfection. At 3 weeks after treatment, longer-term clearance dynamics are captured. At 9 weeks after treatment, reinfection was captured, but failed clearance could not be distinguished from rapid reinfection. Therefore, frequent sampling is required to understand these important epidemiological dynamics.

## Teaser

The impact of treatment for schistosomiasis in high-endemicity settings is short-lived because treatment is far less efficacious than the World Health Organization–recommended Kato-Katz method would suggest, and reinfection occurs more rapidly than is detected by current surveillance time frames.

More than 240 million people live with schistosomiasis, disproportionately affecting those in low- and middle-income countries [1]. Humans are infected through contact with contaminated freshwater, where infective cercariae burrow through the skin. Once matured, *Schistosoma mansoni* reside in the mesentery venules, forming sexually reproducing dioecious pairs and remaining directly unobservable for up to 40 years, producing hundreds of eggs daily [2]. While the worms cause little morbidity, eggs not expelled through defecation remain lodged in the intestines and liver, causing inflammation. Chronic infections can result in varying levels of morbidity, from abdominal pain and diarrhea to enlarged liver and spleen [3], cancer [4], and death [5].

The 2021–2030 neglected tropical disease (NTD) road map from the World Health Organization (WHO) renews the commitment to eliminating schistosomiasis as a public health problem [1, 6]. Key to this is the use of the anthelmintic praziquantel through mass drug administration (MDA). However, praziquantel acts only on adult worms, leaving juveniles to mature within the human host, does not prevent reinfection [7, 8], and is rarely 100% efficacious even against adult worms [9]. Infection after treatment could therefore be due to (1) inadequate clearance, (2) new infections since treatment (reinfection), (3) juveniles at treatment reaching maturation, (4) transient adult embryostasis [10], or a combination of these factors. In addition, because worms cannot be directly observed diagnosis and surveillance rely on proxy measures of infection without clear guidance on time scales of surveillance needed to characterize infection dynamics. Disentangling these factors is important for understanding treatment impact and required frequency of intervention.

Historically, the WHO recommended Kato-Katz thick smears for diagnosis and mapping of *S. mansoni*. Despite widespread use, measuring only eggs, this has proved to be an imperfect method for estimating total worm burden and is plagued by low sensitivity and high variability within individuals [11]. The urine-based point-of-care circulating cathodic antigen (POC-CCA) test is now also endorsed by the WHO. It detects a regurgitated antigen from any feeding schistosome worms, therefore detecting juveniles and reproductively quiescent pairs in addition to egg producing pairs detectable with the Kato-Katz method. The improved sensitivity of the POC-CCA test is particularly evident after treatment and in settings with low infection intensity, such that its use greatly increased global schistosomiasis prevalence estimates [12].

The POC-CCA test has traditionally been scored as negative, trace, +, ++, or +++ (hereafter POC-CCA+) providing a semiquantitative indication of infection intensity. However, ongoing debate about the interpretation of trace results as negative or positive [8, 13–15] has resulted in significantly divergent prevalence estimates [8]. A new scoring method, the G-Score [16], uses preprinted cassettes for direct comparison, with scores from G1 (negative) to G10 enabling more systematic scoring, with reduced interobserver variation and higher resolution. Studies have sought to compare the sensitivity and specificity of POC-CCA tests and the Kato-Katz method [11, 17], and more recently the G-Score [18]. However, because worm burden cannot be directly observed, there is no diagnostic reference standard.

Latent class analyses are a heterogeneous set of statistical models, of which hidden Markov models (HMMs) are a subset dealing with categorical latent (unobserved) variables. Valuable when there is no reference standard, the “true” infection status of an individual is a latent variable probabilistically estimated with the diagnostics considered imperfect estimators of this status, such that each additional diagnostic adds information. This is beneficial, for example, when a diagnostic lacks sensitivity or specificity but can be complemented by an alternative imperfect diagnostic—as is the case for the Kato-Katz and POC-CCA diagnostics. Such analyses have been used to investigate the relative accuracy of Kato-Katz thick smears, POC-CCA tests, and other diagnostics [8, 13–15] at fixed time points and across different endemicity areas; to date, however, the question of clearance and reinfection has not been addressed with a latent class analysis framework. 

Previous modeling efforts have also failed to overcome 2 distinct challenges. First, previous studies using latent class analyses have made governing assumptions regarding trace score interpretation, considering trace scores as positive or negative from the outset, presenting both versions of the results separately [13, 14], or resorting to brute-force methods that do not reflect the biological processes generating the data, instead taking a roundabout route to recapitulate the observed infection dynamics [8]. Second, the differences between egg- and antigen-based estimates of clearance and reinfection rates have not been truly quantified. In addition to these challenges, the robust analysis of G-Score performance is in its infancy.

Here, we present an HMM that identifies the relationship between the quantitative Kato-Katz thick smears and the semiquantitative POC-CCA tests without making governing assumptions about trace score interpretation. We quantify the rates of unobservable clearance and reinfection after treatment by tracking the probability of infection at the individual level over 6 months after treatment. We incorporate 3 diagnostic methods, including the new G-Score method, assessing the suitability of different diagnostic methods and quantifying the impact of treatment.

## METHODS

### Sample and Data Collection

Data were collected as part of a longitudinal study at Bugoto Lake View Primary School, Mayuge District, Uganda, from 210 children with even sex distribution (Supplementary Figure 1), aged 6–14 years. Stool and urine samples were collected before observed treatment with 40-mg/kg praziquantel given after food, in September 2017, and then 3 weeks (October 2017), 9 weeks (December 2017), and 6 months (March 2018) after treatment. At each time point, stool samples were collected over 3 days for duplicate Kato-Katz thick smears (6 slides). A single urine sample was collected from each child before treatment and at 3 weeks and 6 months after treatment; at 9 weeks after treatment, a smaller subsection of this cohort (55 children) provided urine samples. The implication of this sampling schedule, and incomplete data for some children, on model fitting is described below. All urine samples were processed for POC-CCA tests (Rapid Medical Diagnostics; batch no. 180314027l; expiration March 2020) and scored with the POC-CCA+ and the G score [16]. Further details are in the Supplementary Materials.

### The Model

**Figure 1.**
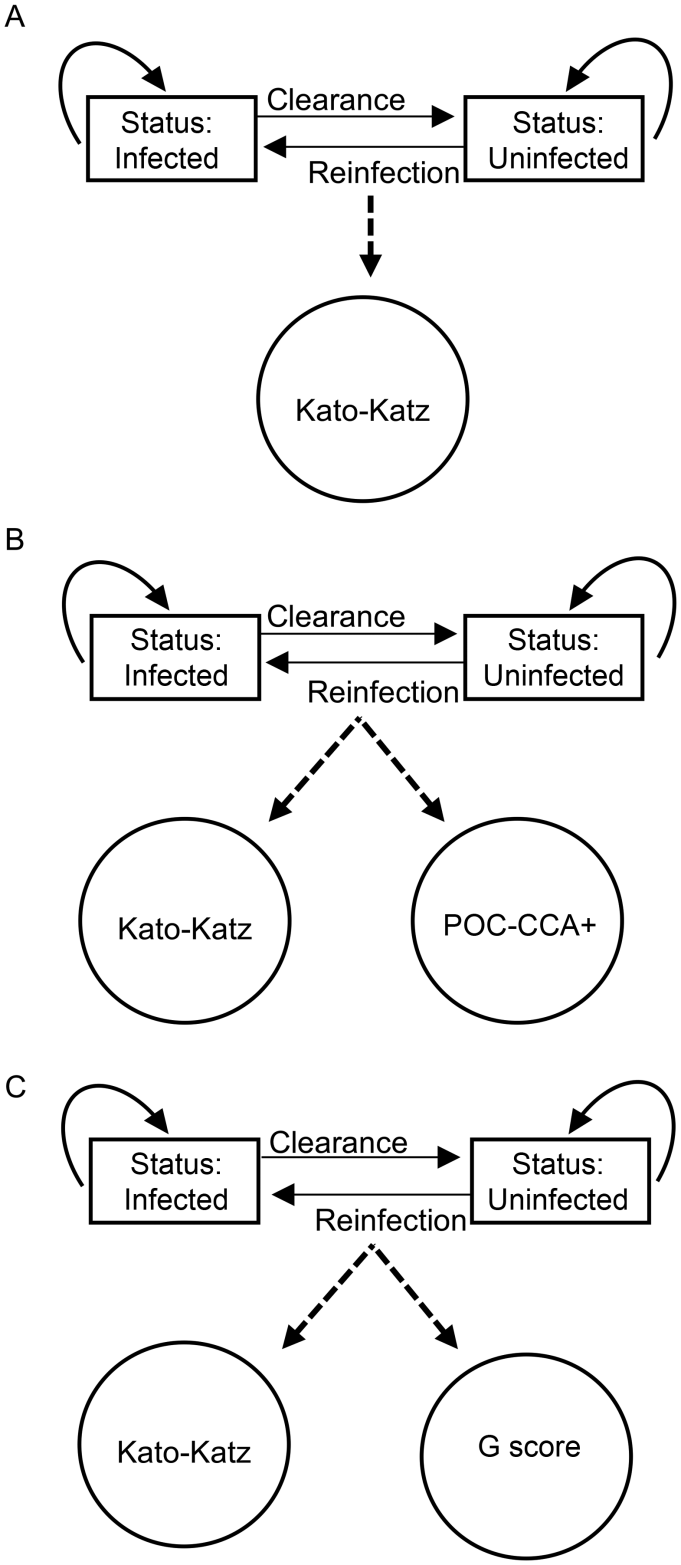
The 3 parameterizations of the hidden Markov model framework. Rectangles represent biological processes that are unobserved and give rise (*dotted lines*) to the observable processes in circles (ie, diagnostic data). Solid arrows indicate the direction of movement between time steps: remain infected, remain uninfected, become uninfected (clearance), or become infected after clearance (reinfection). *A,* Kato-Katz data only. *B,* Kato-Katz and POC-CCA+ data. *C,* Kato-Katz and G-score data.

To estimate the unobservable clearance and reinfection dynamics of *S. mansoni* after treatment, we developed a discrete time stochastic HMM, using the diagnostics data from our 4 time points. We fit 3 versions of the model, using (1) Kato-Katz data alone, (2) Kato-Katz and POC-CCA+ data, and (3) Kato-Katz and G-Score data (Figure 1). The Bayesian framework can make use of incomplete data, imputing missing values probabilistically as a function of a prior distribution on the data and the data itself. In addition, the parameter sampling process uses information from the neighboring time steps, further assisting the model-fitting process in the absence of data (of particular importance in this case for the 9-week time point). Data from all the children were therefore included, even if incomplete, with missing data inferred. Imputed values did not contribute to the likelihood (fitting) of the model (see the Supplementary Materials for posterior distributions of all estimated parameters; Supplementary Figures 3–8).

HMMs consist of unobservable (“hidden”) states, here the infection status of each individual (either infected or uninfected), and observable processes, here the outcomes of the different diagnostics, which are driven by the hidden states. Individuals with undetectable infections (negative by all diagnostic methods) were considered uninfected in the model, because status was estimated based on the diagnostic data at each time point. Between each time step, an individual could remain in the same state (ie, remaining infected) or could switch to another state, for example, an infected child becoming uninfected (Figure 1). From the diagnostic data and through the iterative sampling process used to fit the model (JAGS; implemented in the R version 4.0.2 *runjags* package [19, 20]), the individual infection state at each time point was inferred. The probability of switching state can be interpreted as the clearance and reinfection probabilities respectively. We assumed no reinfection between treatment and 3 weeks (Supplementary Figure 4 and Supplementary Materials).

The relationship between the observation process (the diagnostic results) and the hidden infection states was modeled as follows. For the Kato-Katz method, truly uninfected individuals must have zero counts (assuming 100% specificity). Infected individuals have a true infection intensity drawn from a gamma-distributed population-level mean at baseline, with an autoregressive random walk component used to estimate true intensity at subsequent time points. We assumed an individual’s true infection intensity to be linearly correlated with this population mean, with the overdispersion of the Kato-Katz counts modeled with a negative-binomial distribution, accounting for interday and intraday variation. The variance in each step of the autoregressive component was scaled to account for the length of the time step and the effect of treatment (Supplementary Materials).

When modeling the POC-CCA+ and G Scores, we assumed the true antigen value to be related to true infection intensity following a logistic function. The true antigen values range from 0 to 4 when using POC-CCA+ (representing results from negative to +++), while they range from 0 to 9 when using G Scores (representing scores from 1 to 10). The estimated values of these 2 diagnostics were allowed to vary around the true antigen value following a gaussian (normal) distribution. The variance was calculated from the observed data and fixed in the model.

### Role of the Funding Sources

The funding providers had no role in study design, data collection, analysis, interpretation, or report writing. The corresponding authors had full access to all study data and had final responsibility for the decision to submit for publication.

### Ethical Clearance

Ethical approval for data collection was granted from the Vector Control Division Research Ethics Committee (no. VCDREC/062), Uganda National Council of Science and Technology (no. UNCST-HS 2193), and the University of Glasgow Medical, Veterinary and Life Sciences Research Ethics Committee (no. 200160068). Signed or thumb-printed informed consent was obtained before data and sample collection from the parent or legal guardian for all recruited children, and informed assent was obtained from all children aged ≥8 years.

## RESULTS

### Population Characteristics and Pretreatment Prevalence

**Figure 2.**
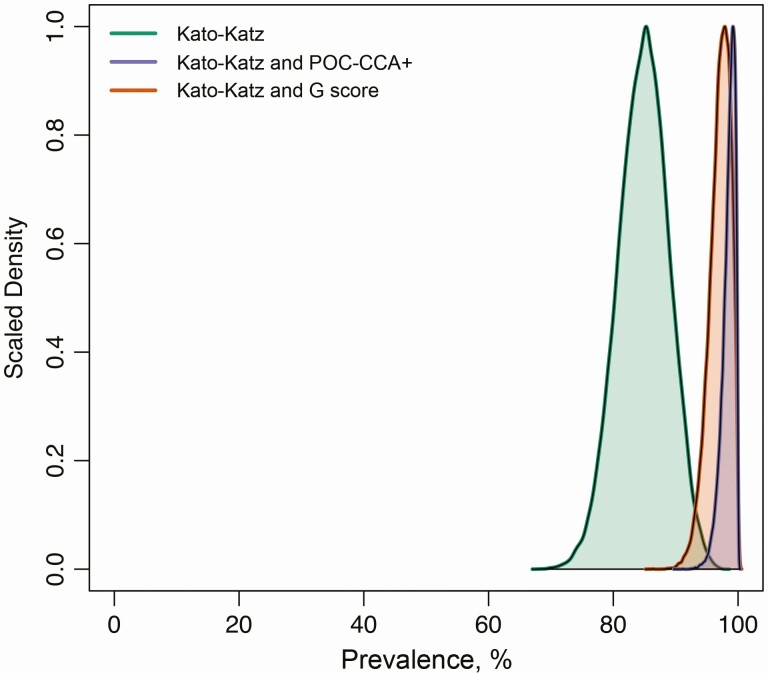
Pretreatment prevalence of *Schistosoma mansoni* estimated by each model. The model using Kato-Katz data only is displayed in green; that using Kato-Katz and POC-CCA+ data, in purple; and that using Kato-Katz and G-score data, in orange.

The model using Kato-Katz data alone model estimated the baseline prevalence of *S. mansoni* to be 85% (95% credible interval [CI]: 79%–92%) (Figure 2). The Kato-Katz and POC-CCA+ model provided higher estimates of 99% (95% CI: 97%–100%) similar to the Kato-Katz and G-score model, at 98% (95% CI: 95%–100%) (Figure 2).

### Treatment Efficacy and Reinfection Rates

**Figure 3.**
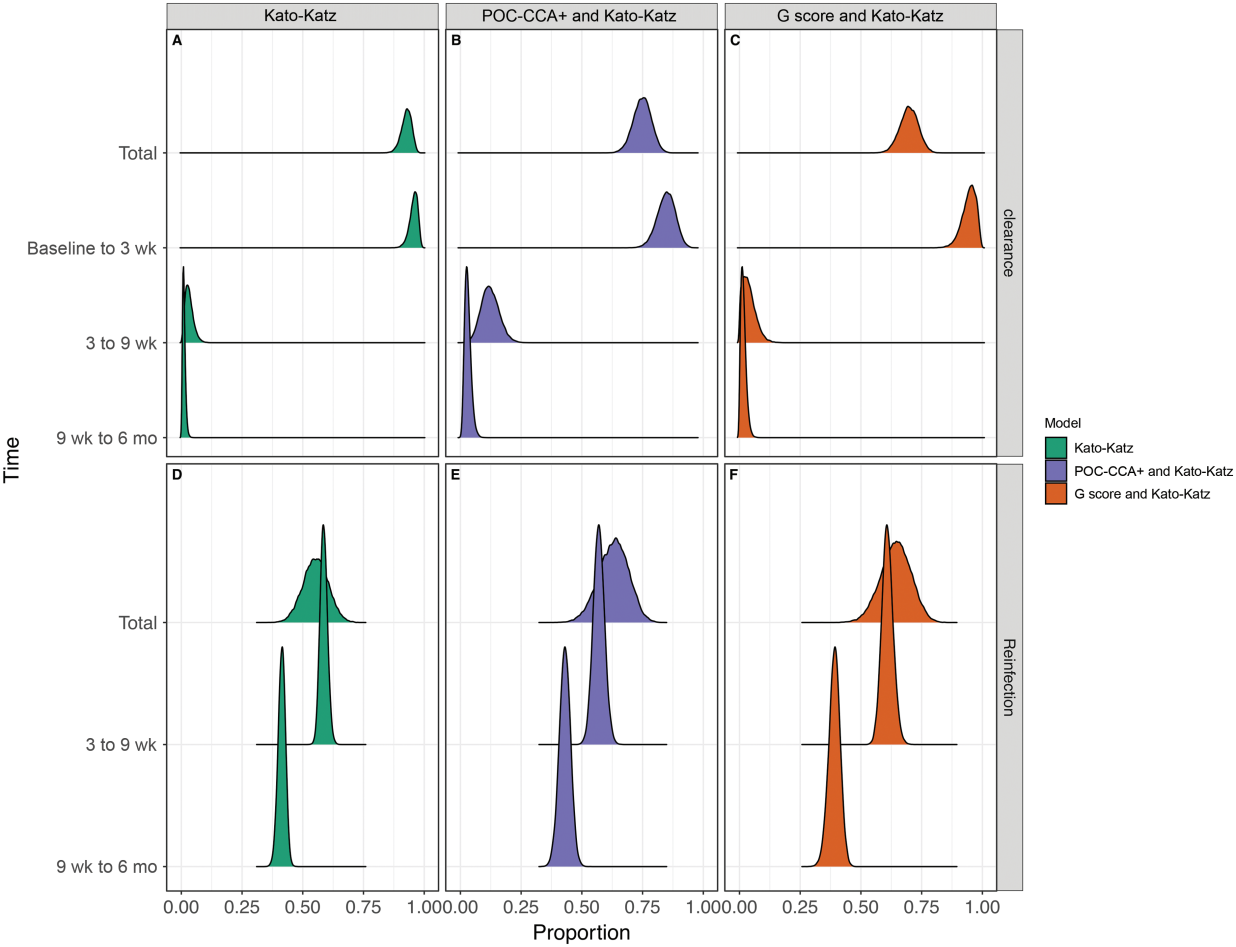
Clearance and reinfection dynamics of *Schistosoma mansoni* after treatment. The first row in each panel (*A–F*) is the posterior distribution of the total additive clearance (*A–C*) or reinfection (*D –F*) after 6 months of surveillance. Total clearance is shown as a proportion with respect to the number of infected children at the beginning of the study. Total reinfection is shown as a proportion with respect to the number of individuals whose infection cleared. The following rows portray the temporal dynamics from baseline to 3 weeks, from 3 to 9 weeks, and from 9 weeks to 6 months. The proportion of clearance at each time step is shown as a proportion of total clearance, whereas reinfection is the proportion of children who were reinfected after clearance of infection in the previous time step. The colors follow those in Figure 2: the model using Kato-Katz data only is shown in green, the model using Kato-Katz and POC-CCA+ data in purple, and the model using Kato-Katz and G-score data, in orange.

We estimated the proportion of infected individuals who cleared infection (Figure 3A–3C) and the proportion of those who cleared infection, but became reinfected within 6 months (Figure 3D–3F). Using Kato-Katz data alone, 93% (95% CI: 88%–96%) of the children were estimated to clear infection during the 6 months of surveillance. This estimated clearance rate decreased when both Kato-Katz and POC-CCA results were considered, with a mean of the posterior distribution of 72% (95% CI: 64%–79%) clearing infection with the Kato-Katz and POC-CCA+ model, and 65% (57%–73%) with the Kato-Katz and G-Score model. Estimates from all 3 models indicate that most clearance occurred within 3 weeks after treatment (Kato-Katz–only model, 96% [95% CI: 92%–98%]; Kato-Katz and POC-CCA+ model, 78% [69%–86%]; and Kato-Katz and G-Score model, 93% [85%–99%]). For those whose infection had cleared, 55% (95% CI: 45%–65%) were estimated to be reinfected within 6 months by the Kato-Katz model, while this estimate increased to 63% (50%–74%) with the Kato-Katz and POC-CCA+ model and 64% (51%–76%) with the Kato-Katz and G-score model. All models agreed that more than half of all reinfections had already occurred within 9 weeks after treatment; however, reinfections continued to occur for the remaining 4 months.

### True Schistosomiasis Prevalence Over Time

**Figure 4.**
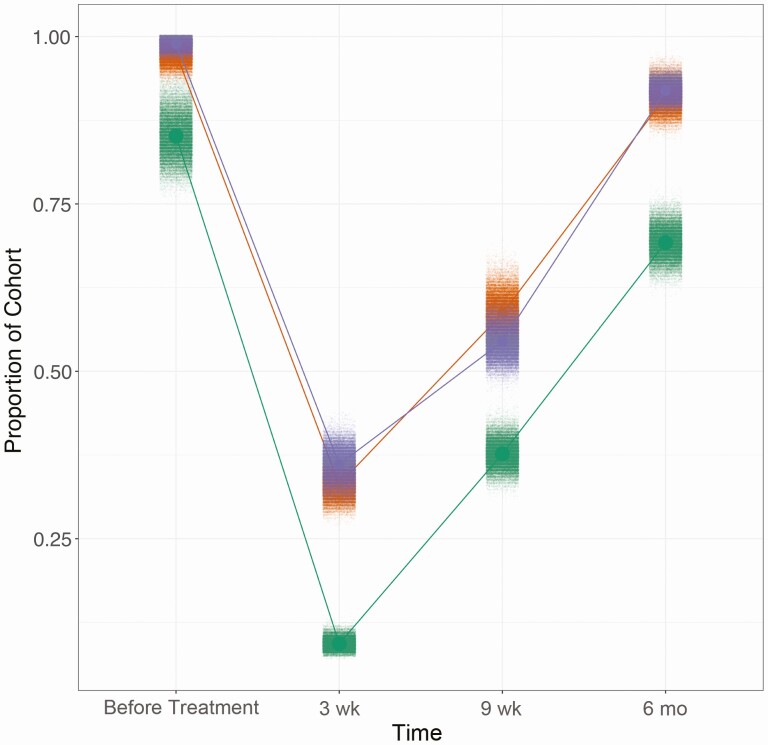
Disease prevalence displayed as proportion of the cohort infected at each time point, estimated by each model. The clouds of each color correspond to draws from the posterior distribution for each time point in each model. Green represents the model using Kato-Katz data only; purple, the model using Kato-Katz and POC-CCA+ data; and orange, the model using Kato-Katz and G-score data.

The above-described dynamics result in the infection prevalence at each time point shown in Figure 4. At 3 weeks after treatment, the prevalence estimated by the Kato-Katz method alone was 9% (95% CI: 8%–11%). By 6 months after treatment the Kato-Katz–only model estimated this to have returned to 69% (95% CI: 64%–74%). Alternatively, the additional use of POC-CCA data estimated less efficacious treatment. Prevalence estimates when the models included POC-CCA+ or G-score data were 36% (95% CI, 31%–41%) and 33% (30%–37%), respectively, at 3 weeks after treatment. This was followed by increases in prevalence by 6 months after treatment to 92% (95% CI: 90%–94%) and 91% (87%–95%), respectively.

## Discussion

We quantified the temporal dynamics of *S. mansoni* clearance and reinfection over 6 months after praziquantel treatment. Our model framework improves on existing models by making no assumptions regarding the interpretation of trace POC-CCA scores. Our model was also suitable for the inclusion of G-Score data through a simple modification. A benefit of this approach is the flexibility imparted by the logistic function. If there were a linear relationship between the POC-CCA scores and infection intensity, the logistic function would take on a linear form. Furthermore, because the parameters of the logistic function estimated by the model are done so with regard to the data the model is fitted to, this framework can readily be applied to data from low-prevalence/low-intensity locations. 

Latent class analyses are often used in diagnostic sensitivity and specificity analyses but are limited in their practicality because of the artificial positivity thresholds implemented for trace results [13]. Our improved framework therefore lends itself, with some modification, to such analyses where it could provide more direct and less subjective insight into diagnostic performance, and interpretation of the debated trace/G2 or G3 test results.

It is notable that these POC-CCA tests were conducted in accordance with the manufacturer’s guidelines at the time, with 2 drops of urine rather than the single drop and chase buffer, as was historically used. There is evidence that this new method is less specific than the original method and can lead to a significant number of false-positives [21]. However, we did not find concerning evidence of this in our data (Supplementary Figure 2 and Supplementary Materials). In short, the observed score variation between time points is accounted for by the stochastic score allocation in the model and the structure of the model itself, where the use of multiple diagnostics provides an improved indication of infection status compared with a single diagnostic (Supplementary Figures 9–13).

Treatment efficacy is historically quantified by comparing egg counts before and after treatment. Our models indicate that this approach overestimates treatment efficacy and inadequately identifies and quantifies reinfection, with major implications for the timing of MDA. The WHO guidelines recommend annual praziquantel in high-risk communities [22]. Having shown reinfection within 9 weeks after treatment, our work indicates that even if annual treatment is efficacious, the prolonged time between treatments may not be sufficient to reduce the FOI. 

Our study was performed 4 months after MDA, with only the recruited cohort being treated at this time, and therefore it is unlikely this would affect community-wide force of infection. However, coverage in this community is known to be low [23], suggesting that community-wide treatment may not be significantly more effective than a school-based program unless coverage is increased. If so, it is likely that the FOI is not decreasing in this area with MDA. Furthermore, monitoring and evaluation is an important but often overlooked aspect of NTD control, but when it does occur it most often happens alongside treatment. It is therefore clear from our results that key insights into the dynamics of *S. mansoni* (re)infection are being missed. Sampling 3 weeks after treatment is sufficient for the characterization of treatment efficacy (Figure 3A–3C), and sampling 9 weeks after treatment successfully captures reinfection dynamics, particularly when using the G-Score method. However, sampling only at 9 weeks after treatment would provide an insufficient estimate of clearance.

The differences in epidemiological dynamics estimated between the Kato-Katz–only model and the Kato-Katz and POC-CCA models can be explained by several hypotheses. The reduced rate of clearance in the Kato-Katz and POC-CCA models is likely explained by non–egg-producing worms or low levels of egg production. Surviving adults still produce detectable antigens; however, treatment-induced reproductive quiescence has been seen in *Onchocerca* and *Ascaris* [24, 25] and is supported by experimental evidence in *S. mansoni* [26]. In addition, juveniles present before treatment are not susceptible to praziquantel, and although not reproductive, produce detectable antigens. Third, in high-endemicity areas, new juveniles could be contributing to the POC-CCA–positive results after treatment. In all 3 instances, individuals could be egg negative, but POC-CCA positive at 3 weeks, and could resume or start egg production a few weeks later ,contributing to morbidity and transmission rates [7]. The difference between the models incorporating Kato-Katz and POC-CCA data and the Kato-Katz–only model at 3 weeks after treatment indicates the presence of adult and/or juvenile worms, but no detected eggs, in approximately 20%–30% of the children.

Rapid reinfection also shows that improving treatment accessibility and equitability [27] to demographic groups other than School-aged children (SAC) is crucial [28]. Adults are only included in MDA in instances of high SAC prevalence, and coverage in adults remains poor [29], despite evidence of their contribution to transmission [23, 28]. Similarly, until a safe and efficacious pediatric praziquantel formula is in development [30], pre-school-aged children are rarely included in treatment programs despite harboring heavy infection loads and contributing to transmission and being at risk of morbidity [31, 32].

A secondary treatment within weeks of the primary treatment to target the surviving juveniles has been suggested as a solution to rapid resurgence. Though shown to be effective [33], innate resistance has been seen in Mayuge District since 2005, around the interception of MDA in Uganda [34]. Thus, increasing treatment frequency may increase selection for resistance, and indeed increase the pressure for an already limited resource [1]. The presence of treatment resistance accentuates the importance of co-occurring mitigation strategies, such as WASH (Water, Sanitation and Hygiene), into NTD management. WASH has been shown to reduce the odds of infection [35]; however, overall, empirical research into the implementation, timing, and success of WASH is still lacking [36].

To conclude, we present an HMM framework that makes no assumptions about the interpretation of trace scores. Using Kato-Katz and POC-CCA data, including the recently developed G-Score method for POC-CCA tests, we quantified the temporal dynamics of clearance and reinfection of *S. mansoni* after treatment in a high-endemicity area. Our results raise questions regarding treatment efficacy, the timing of surveillance, and what type of infection the POC-CCA test is detecting. Quantifying the probability of infection for those who receive POC-CCA+ trace or G2/G3 scores will be integral to answering these questions.

## Supplementary Data

Supplementary materials are available at *Clinical Infectious Diseases* online. Consisting of data provided by the authors to benefit the reader, the posted materials are not copyedited and are the sole responsibility of the authors, so questions or comments should be addressed to the corresponding author.

ciab679_suppl_Supplementary_Materials

## Notes

***Acknowledgments.*** We are grateful to the District Vector Control Officer, Juma Nabonge; the head teacher and teachers, particularly Nankwanga Prossy at Bugoto Lake View; the village health team; and the village chairpersons for their efforts in mobilizing the community for this study. We are most grateful to the children who provided the samples and data for this study. We also thank Lisette van Lieshout PhD for making the G-Score cassettes available for use.

***Disclaimer.*** The funders had no role in the study design, data collection and analysis, decision to publish or preparation of the manuscript

***Financial support.*** This work was supported by the European Research Council (starting grant SCHISTO_PERSIST_680088 to P. H. L. L.); Wellcome (grant 204820/Z/16/Z to P. H. L. L.); the Engineering and Physical Sciences Research Council (grants EP/T003618/1 to J. M. P. and P. H. L. L. and EP/R01437X/1 to principal investigator J. Cooper award EP/R01437X/1 was only awarded to PI J. Cooper, University of Glasgow, not JMP.); and the Medical Research Council (grant MR/P025447/1 to P. H. L. L.).

***Potential conflicts of interest.*** A. M., A. N., A. M., D. A., F. B., A. A., and A. W. and received per diems while performing field work supported by the European Research Council, which also paid a salary to R. F. as a research assistant and whilst registered as a PhD student funded research costs and a salary for C. L. F. (grant SCHISTO_PERSIST 680088 for all). E. M. T. received 10% of their wage from the Medical Research Council (grant awarded to P. H. L. L.). L. V. C. was awarded a studentship from the University of Glasgow (Institute of biodiversity, animal health & comparative medicine [IBAHCM] stipend 144536) to cover the cost of a 3-year PhD stipend and university fees. P. H. L. L. was paid to conduct this work by the European Research Council (starting grant), Wellcome (returners grant), the Engineering and Physical Sciences Research Council, and the Medical Research Council. All other authors report no potential conflicts. All authors have submitted the ICMJE Form for Disclosure of Potential Conflicts of Interest. Conflicts that the editors consider relevant to the content of the manuscript have been disclosed.
