# Supplementary material for: Reconciling Egg- and Antigen-Based Estimates of Schistosoma mansoni Clearance and Reinfection: A Modeling Study
Source: Clin Infect Dis. 2021 Aug 6;74(9):1557–63. doi: 10.1093/cid/ciab679 (PMC9070857; doi:10.1093/cid/ciab679)
Supplement: ciab679_suppl_Supplementary_Materials [file ciab679_suppl_supplementary_materials.docx]

Supplementary Material

**Reconciling egg- and antigen-based estimates of *Schistosoma mansoni* clearance and reinfection; a modelling study**

Jessica Clark^1^, Moses Arinaitwe^2^, Andrina Nankasi^2^, Christina L. Faust^1,^, Adriko Moses^2^, Diana Ajambo^2^, Fred Besigye^2^, Alon Atuhaire^2^, Aidah Wamboko^2^, Lauren V. Carruthers^1^ , Rachel Francoeur^1,3^, Edridah M. Tukahebwe^2^, Joaquin M. Prada^4*^ & Poppy H. L. Lamberton^1,2*^

1. Wellcome Centre for Integrative Parasitology, Institute of Biodiversity, Animal Health & Comparative Medicine, Glasgow, UK
2. Vector Control Division, Ministry of Health, Uganda
3. Faculty of Medicine & Life Sciences, University of Chester, Chester, UK
4. Faculty of Health & Medical Sciences, University of Surrey, Guildford, UK

*Joint senior author

Here, we present the details of additional data collection protocols and the Hidden Markov Model framework to accompany the description provided in the main text.

**Data**

Data were collected in Mayuge District, which is classified by the WHO as high endemicity for *S. mansoni*. At the time of data collection, it had received 14 years of MDA. Thirty SAC from each age between six and 14 (270 total) with an even sex distribution were recruited in March 2017. After data cleaning and sample assessment, in this analysis, we focus on 210 of these children. 55 of these children were also part of a separate cohort study conducted in parallel, where they were measured twice a week for 6 months. We have Kato-Katz data at the nine-week post treatment timepoint for the full cohort including the 55, but it is for these 55 children that we also have nine-week POC-CCA data. Stool samples were provided and read by experienced, skilled technicians. Urine was collected at the same time and stored first at room temperature then frozen before a POC-CCA was evaluated by one constant reader and one rotating second reader. After pre-treatment sample collection, all children were fed before observed treatments of 40mg/kg praziquantel were administered according to weight, rather than dose pole, alongside albendazole, and where necessary malaria treatment 24 hours later.

Figure S 1. The number of children providing samples at each time point disaggregated by sex.

In light of the evidence to suggest that POC-CCAs can produce false positives when using the new manufacturers protocol (as performed here), we have plotted out the flow of POC-CCA+ scores over time by individual.

Figure S2: Alluvial plot showing the flow of POC-CCA+ scores over time.

This shows two children who go from having a score of ++ (blue) to negative (red) between nine-weeks and six-months. It is possible that these are due to a lack of sensitivity at six-months post treatment, or a lack of specificity at nine-weeks post treatment, however both of these types of errors will have been accounted by the model. The instances where children’s scores differ by just one score can be explained as much by reader variability as by diagnostic performance. For example, there are a handful of children who have scores of Trace or + at nine-weeks and go to negative and Trace respectively at six-months. This level of reader variability or imperfect diagnostic performance is accounted for in the model as is described in the methods. Additionally, the use of both diagnostics in the model can limit the impact of this error; if the POC-CCA is erroneously and inconsistently considering individuals positive (for example, negative pre-treatment, positive three-weeks post treatment but negative again at nine-weeks), but negative by Kato-Katz at all timepoints, the model is likely to infer that person as negative.

**Model Details**

The model is a Hidden Markov Model, linking together a latent (hidden) infection status of an individual and the measured diagnostics (such as Kato-Katz and POC-CCA). Individual’s status over time changes based on the clearance and reinfection probabilities. Moreover, an auto-regressive type 1 model (AR1) is used to track variation of infection burden over time.

**Infection Status**

We draw on previous zero-inflated model frameworks [1, 2] with the ability to distinguish between individuals who are truly infected and those who are either uninfected or undetectably infected. The status of individual $i$ at time $t$ is denoted

$${Status}_{i,t}=\left\{ \begin{aligned} 0 \\ 1 \end{aligned} \right.\begin{aligned} uninfected\vee undetectable infection \\ infected \end{aligned}$$

The status of individual *i* at time *t* can change or remain the same, depending on their current status. For each individual at each time-point we estimate whether they cleared infection (if ${Status}_{i,t}=1$) or become infected (if ${Status}_{i,t}=$0), based on the clearance and re-infection probability at that time-point

$${Cleareance}_{i,t} \sim Bernoulli({P(Clearance}_{t}))$$

and

$${Reinfection}_{i,t} \sim Bernoulli({P(Reinfection}_{t}))$$

Such that

$${Status}_{i,t}={Status}_{i,t-1}{Clearance}_{i,t-1}+{1-Status}_{i,t-1}{Reinfection}_{i,t-1}$$

For clarity, the main text presents the model results in terms of the proportion of children infected at each time point, derived from the proportion of children who were infected who then cleared infection between each timepoint, and the proportion of children who had cleared infection in the previous time point, but had become reinfected by the next. The posterior distributions for the clearance and reinfection dynamics were unimodal as shown in figures S1 and S2 respectively. Maturation to adult reproducing worm in the human host takes 5-7 weeks. Thus, any child excreting eggs three-weeks post-treatment but egg negative at baseline, had to have been infected at baseline.

Figure S3. The model posterior distributions from each model for the probability of Schistosoma mansoni clearance between each timestep (Kato-Katz alone in green, Kato-Katz and point-of-care circulating cathodic antigen (POC-CCA) G-Score in orange and Kato-Katz and POC-CCA CCA+ in purple).

Figure S4. Posterior distributions from each model for the probability of Schistosoma mansoni reinfection between each time step. Kato-Katz alone in green, Kato-Katz and point-of-care circulating cathodic antigen (POC-CCA) G-Score in orange and Kato-Katz and POC-CCA CCA+ in purple

**Infection Intensity**

Infection intensity for individual $i$ at time $t$ (given by $\lambda_{i,t}$) was modelled with a Gaussian random walk where $\lambda_{i,t}$is dependent on $\lambda_{i,t-1}$. Thus $\lambda_{i,1}$is given by

$$\lambda_{i,1}=\left\{ \begin{aligned} 0 \\ Gamma\left( \alpha,\beta\right) \end{aligned} \right.$$

Where the top value refers to those who are uninfected/ infected with undetectable infections, and the lower to those with ${Status}_{i,t}= 1$, With the shape $\alpha$ and rate $\beta$ parameters estimated by the model (Figure S3).

Figure S5. The posterior distributions of the shape and rate parameters of the gamma distribution from which the true Kato-Katz counts at baseline were drawn, for each model. Kato-Katz alone in green, Kato-Katz and point-of-care circulating cathodic antigen (POC-CCA) G-Score in orange and Kato-Katz and POC-CCA CCA+ in purple.

The following timepoints for uninfected/ non-detectable, and infected individuals are given by

$$\lambda_{i,2:4}=\left\{ \begin{aligned} 0 \\ N\left( \lambda_{i,t-1},\tau_{t} \right) \end{aligned} \right.$$

Where the top value refers to those who are uninfected/ infected with undetectable infections, and the lower to those with ${Status}_{i,t}= 1$, and where $\tau_{t}$ represents the time-dependent inverse variance (precision) estimated by the model at each time point. $\tau_{t}$ was scaled to account for the differences in time between each sampling event. Because the effect of treatment on the variation of egg counts over time is unknown, we assume the variation scales inversely proportionally to the time between timepoints (i.e. largest variance from baseline to three-weeks post-treatment and smallest variance from nine-weeks to six-months, where we expect the variance to build up and stabilize). We also assume 100% specificity such that a truly uninfected individual cannot possibly have eggs.

**Kato-Katz Likelihood**

To account for the variation inherent in Kato-Katz measurements, we use a Gamma-Negative Binomial process to model the likelihood. Each individual $i$ presented a repeat set of samples $r$ at time $t$ giving

$${Kato-Katz}_{i,t,r}=\left\{ \begin{aligned} 0 \\ NB\left( \frac{\alpha_{2}}{\lambda_{i,t-1}+\alpha_{2}},\alpha_{2} \right) \end{aligned} \right.$$

Where the top value refers to those who are uninfected/ infected with undetectable infections, and the lower to those with ${Status}_{i,t}= 1$. Parameter $\alpha_{2}$ is a shape parameter estimated by the model, but different to that of the gamma distributed shape parameter above (Figure S4).

Figure S6. The posterior distributions from each model, for the shape parameter of the Gamma-Negative Binomial process of the Kato-Katz data likelihood. Kato-Katz alone in green, Kato-Katz and point-of-care circulating cathodic antigen (POC-CCA) G-Score in orange and Kato-Katz and POC-CCA CCA+ in purple

**Point-of-care circulating cathodic antigen (POC-CCA) Likelihood**

It is plausible that the higher the infection intensity, the more probable it is that an individual will test positive by POC-CCA. This could be a linear relationship, or it could be sigmoidal, however this relationship has not previously been deduced. To allow for this flexibility, we use a logistic function to describe the relationship between infection intensity, and the probability of scoring on a POC-CCA. The numerator of the logistic function is the maximum value, reflecting the highest POC-CCA scores. The logistic growth rate $-k$ and the sigmoidal intercept $x_{0}$ were estimated by the model. Posteriors are shown below.

**POC-CCA+**

The POC-CCA+ is measured semi-quantitatively from negative, to +++. We transform these into 0 (negative), 1 (trace), 2 (+), 3 (++) and 4 (+++), such that the numerator value is 4 resulting in

$${CCA+}_{i,t}=\left\{ \begin{aligned} \mathcal{N(}0, 3.093451) \\ \mathcal{N}\left( \frac{4}{{{1+(\lambda}_{i,t}-x_{0})}^{-k}},3.093451 \right) \end{aligned} \right.$$

Where the values are then rounded to represent the closest integer value of the CCA+ score. The POC-CCA score for those with $Status=0$ is drawn from a truncated normal distribution (>0) , with a mean of 0. The precision is a fixed value calculated from the distribution of POC-CCA scores across Kato-Katz counts such that even if someone has $Status=0$ they can still be given a score of 1 (Trace).

**G-Score**

The G-Score is measured semi-quantitatively from 1-10. We adjust these to fall from 0-9. The numerator then takes on the value of 9

$${G-Score}_{i,t}=\left\{ \begin{aligned} \mathcal{N(}0, 1.093606) \\ \mathcal{N(}\frac{9}{{{1+(\lambda}_{i,t}-x_{0})}^{-k}},1.093606) \end{aligned} \right.$$

The precision was recalculated accordingly. The $x_{0}$ and $k$ parameters were estimated by the model for each diagnostic and were unimodal (Figure S5 and S6 respectively). Values for all priors are given in table S1.

Figure S7. The posterior distributions for the intercept parameter of the logistic function, as estimated by each model. Kato-Katz and point-of-care circulating cathodic antigen (POC-CCA) G-Score in orange and Kato-Katz and POC-CCA CCA+ in purple

Figure S8. The posterior distributions for the k parameter of the logistic function, as estimated by each model. Kato-Katz and point-of-care circulating cathodic antigen (POC-CCA) G-Score in orange and Kato-Katz and POC-CCA CCA+ in purple

Table S1. Priors and fixed values for model parameters. The priors with regards to the Kato-Katz (KK) data come from Prada et al. (2018) however the point-of-care circulating cathodic antigen (POC-CCA) (CCA+ and G-Score) were modelled differently here and so any parameters pertaining to POC-CCA data were given uninformative priors.

| Parameter | Prior Distribution | values |
| --- | --- | --- |
| Clearance | $Beta\left( \alpha,\beta\right)$ | $\alpha=1,\beta=1$ |
| Reinfection | $Beta\left( \alpha,\beta\right)$ | $\alpha=1,\beta=1$ |
| Prevalence | $Beta\left( \alpha,\beta\right)$ | $\alpha=1,\beta=1$ |
| $\sigma_{t}$ | $Gamma\left( \alpha,\beta\right)$ | $\alpha=0.001,\beta=0.001$ |
| $\kappa$ G-Score | $Gamma\left( \alpha,\beta\right)$ | $\alpha=0.001,\beta=0.001$ |
| $\kappa$ CCA+ | $N\left( \mu,\tau^{2} \right)$ | $\mu=0.0633,\tau^{2}=1/{{0.0098}^{2}}$ |
| Intercept CCA+ | $Uniform\left( a,b \right)$ | $a=0.1395,b=8.5045$ |
| Intercept GS | $Gamma\left( \alpha,\beta\right)$ | $\alpha=0.001,\beta=0.001$ |
| Shape (true KK) | $Gamma\left( \alpha,\beta\right)$ | $\alpha=83.886,\beta=125.703$ |
| Rate (true KK) | $Beta\left( \alpha,\beta\right)$ | $\alpha=47.135,\beta=633.084$ |
| $\alpha_{2}$ | $Gamma\left( \alpha,\beta\right)$ | $\alpha=286.323,\beta=223.465$ |
| $CCA+\tau^{2}$ | NA | 3.093451 |
| $G-Score\tau^{2}$ | NA | 1.083606 |

Given that in the main text, we present the Kato-Katz only model in the absence of POC-CCA data, we also present here, the main figures of the POC-CCA models without the Kato-Katz data. We first show the posterior distributions for clearance and reinfection by time point. Because at nine-weeks there is more Kato-Katz data than POC-CCA data, we can see the removal of this data results in much greater uncertainty (the posterior distributions are wider and messier than their counterparts using POC-CCA and Kato-Katz data). This is particularly evident in the clearance estimate three-to-nine-weeks post-treatment for G-Score and for both models for reinfection nine-week to six-months.

Figure S9 Posterior distributions for the S. mansoni clearance parameter estimates over time. G-Score in orange, POC-CCA+ in purple. We see a particularly poor estimate for clearance using G-Score three-to-nine-weeks in the absence of the Kato-Katz data.

Figure S10 Posterior distributions for the reinfection parameter estimates in the absence of Kato-Katz data. G-Score in orange POC-CCA+ in purple. We see a particularly poor fit nine-week-to-six-months post-treament.

As such, though we have produced the following figures for comparison, this is a poor model fit resulting in somewhat erroneous results.

Figure S11 Prevalence estimates in the absence of the Kato-Katz data. G-Score in orange, POC-CCA+ in purple. The G-Score prevalence estimate is significantly lower in this model than in the model with Kato-Katz

Figure S 12 Clearance (top) and reinfection (bottom) for POC-CCA+ (purple) and G-Score (orange) over time in the absence of Kato-Katz data.


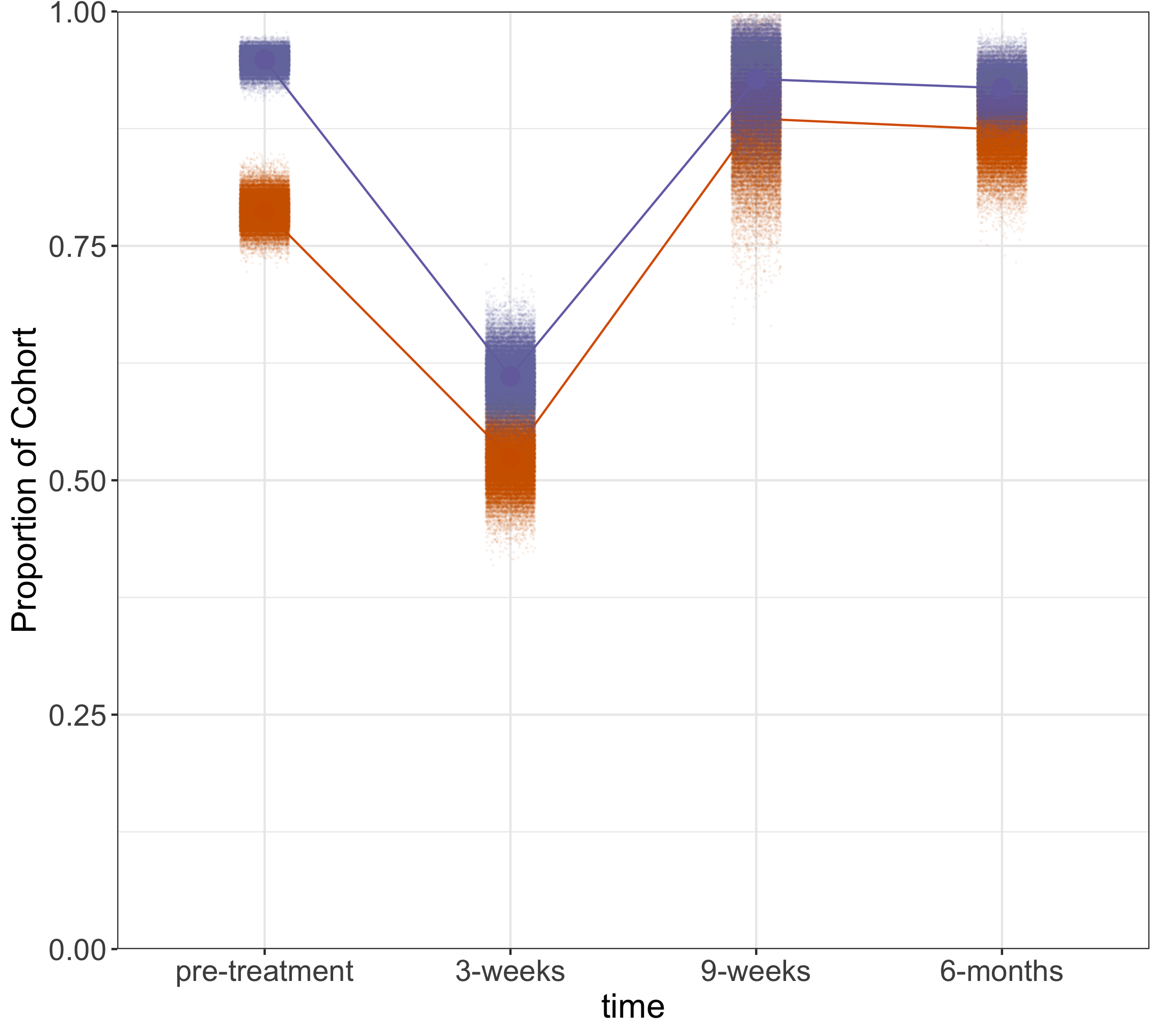


Figure S 13 Prevalence at each time point estimated by POC-CCA+ (purple) and G-Score (orange) in the absence of the Kato-Katz data. We emphasize again, that these models are a poor fit in comparison to the models presented in the main text.

References

1. Atlija M, Prada JM, Gutiérrez-Gil B, et al. Implementation of an extended ZINB model in the study of low levels of natural gastrointestinal nematode infections in adult sheep. BMC Veterinary Research **2016**; 12(1).

2. Prada JM, Touloupou P, Adriko M, Tukahebwa EM, Lamberton PHL, Hollingsworth TD. Understanding the relationship between egg- and antigen-based diagnostics of Schistosoma mansoni infection pre- and post-treatment in Uganda. Parasites & Vectors **2018**; 11(1): 21.
